# Beyond lethal temperatures: Factors behind the disappearance of chum salmon from their southern margins under climate change

**DOI:** 10.1371/journal.pone.0330957

**Published:** 2025-09-10

**Authors:** Yu-Lin K. Chang, Kentaro Honda, Kentaro Morita

**Affiliations:** 1 Application Laboratory, Japan Agency for Marine-Earth Science and Technology, Yokohama, Kanagawa, Japan; 2 Fisheries Resources Institute, Japan Fisheries Research and Education Agency, Sapporo, Hokkaido, Japan; 3 Atmosphere and Ocean Research Institute, The University of Tokyo, Kashiwa, Chiba, Japan; University of Tehran, IRAN, ISLAMIC REPUBLIC OF

## Abstract

The Tone River in Japan represents one of the southern limit distributions of chum salmon (*Oncorhynchus keta*) on the western side of the North Pacific, but the number of adult chum salmon observed here has declined dramatically since 2013 and reached zero in 2024. The factors behind the recent decline of the chum salmon population in the Tone River were investigated by using ocean reanalysis data and a 20-year particle-tracking simulation. Virtual chum salmon fry were released at the mouth of the Tone River in spring each year with six different swimming strategies to evaluate the effects of ocean currents on the population growth rate of salmon. None of the simulated scenarios reproduced the recent decline in the population, regardless of the swimming strategy and addition of lethal temperature thresholds. Instead, the decline in population growth rate appears to be correlated with warming water temperature and reduced zooplankton abundance caused by the northward shifts of the Kuroshio/Kuroshio Extension and Oyashio. Along the coast of northeastern Japan, the warm, nutrient-poor Kuroshio/Kuroshio Extension replaced the cold, nutrient-rich Oyashio on the migration route of chum salmon fry, increasing the water temperature and reducing zooplankton abundance. Partial correlation analysis of the water temperature and zooplankton abundance indicated that the latter was the main influencing factor coherently related to the population growth rate of salmon. The reduced zooplankton abundance would affect the growth and survival of chum salmon fry, which would result in a decline in population growth. The northward shift of the Kuroshio/Kuroshio Extension and Oyashio may continue or return southward depending on the effects of climate change, which will greatly influence the future population growth of chum salmon and whether they come back to the Tone River.

## Introduction

One of the major effects of climate change on organisms is a shift in their geographic distribution, which is particularly pronounced for marine organisms compared with their terrestrial counterparts [1]. Increasing water temperature has driven a northward shift in the distribution of several marine fish species in the Northern Hemisphere [2], which has led to their disappearance from the southern limit of their range. Pacific salmon are not only ecologically important but also a valuable fishery resource and, as cold water species, they are particularly vulnerable to the impacts of climate change [3]. While Pacific salmon are widely harvested along the northern Pacific Rim—comprising the United States (Alaska), Russia, Canada, and Japan—their populations in Japan and Canada, which are at the southern end of their distribution, have seen a marked decline [4]. Although rising water temperature is frequently correlated with northward shifts in marine species, the underlying mechanisms remain surprisingly unclear.

Pacific salmon have an extensive migration range across the North Pacific yet exhibit a remarkable homing ability by returning to spawn in specific natal rivers [3]. Populations at the southern limit of their range face a greater challenge than those at higher latitudes because they not only experience higher temperatures in their natal rivers and adjacent coast but also must migrate longer distances to reach their northern oceanic feeding grounds. Because early life stages are critical to determining fish population dynamics [5], the ability of juvenile salmon to successfully reach these distant foraging areas after descending from their natal rivers is likely to be a key factor influencing the persistence of southern populations.

**Fig 1:**
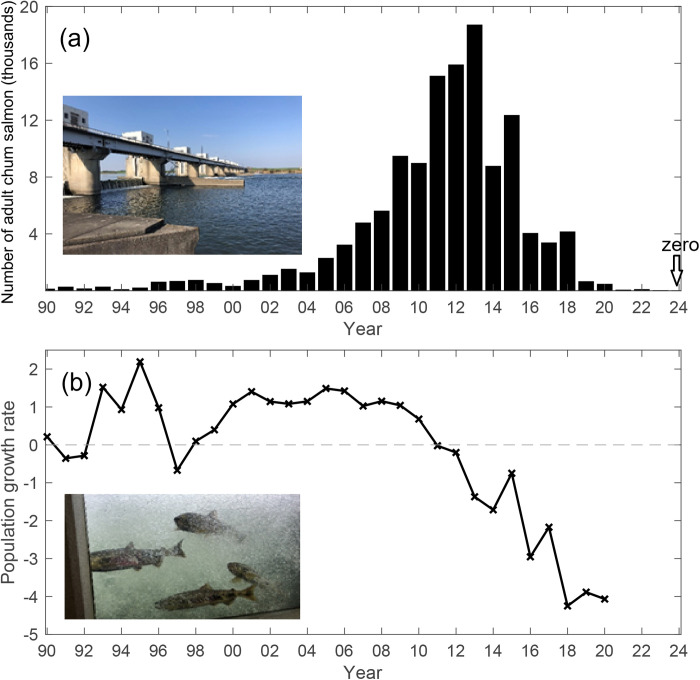
Observed chum salmon at Tone River (a) Number of adult chum salmon (*Oncorhynchus keta*) counted at the Tone River weir 150 km upstream from the river mouth from 1990 to 2024; (b) population growth rate of chum salmon in the Tone River assuming one generation corresponds to four years. Data from the Tone River weir were obtained by the Japan Water Agency (https://www.water.go.jp/kanto/tone/water/fish-data/). Insets show photos of the (a) Tone River weir and (b) chum salmon in the fish counting ladder window taken in November 2018. This figure was created using MATLAB R2021a (http://www.mathworks.com/).

Chum salmon (*Oncorhynchus keta*) are the most widely distributed species of Pacific salmon and spawn in rivers along the northern Pacific Rim [6]. On the eastern side of the North Pacific, the southernmost limit was once the San Lorenzo River, which drains into Monterey Bay in California (36.96° N), but its population has become extinct [7]. The current southern limit on the eastern side has been pushed northward to Oregon (44.6–46.0° N), but these populations are also declining in number [8]. On the western side, the southern limit includes rivers in South Korea and Japan [9,10]. In Japan, the southernmost natal river on the Pacific Ocean side was the Tone River (35.76° N, 140.86° E), which flows through the Kanto Plain, which is one of the most densely populated regions in Japan, within which Tokyo is located. The number of adult chum salmon counted at the Tone River weir increased after 2000 to peak at over 18,000 individuals in 2013 (Fig 1a), which is believed to reflect enhanced natural spawning likely due to improvements in water quality [11]. However, the number of returning adults has declined since then and eventually reached zero in 2024 (Fig 1a).

**Fig 2:**
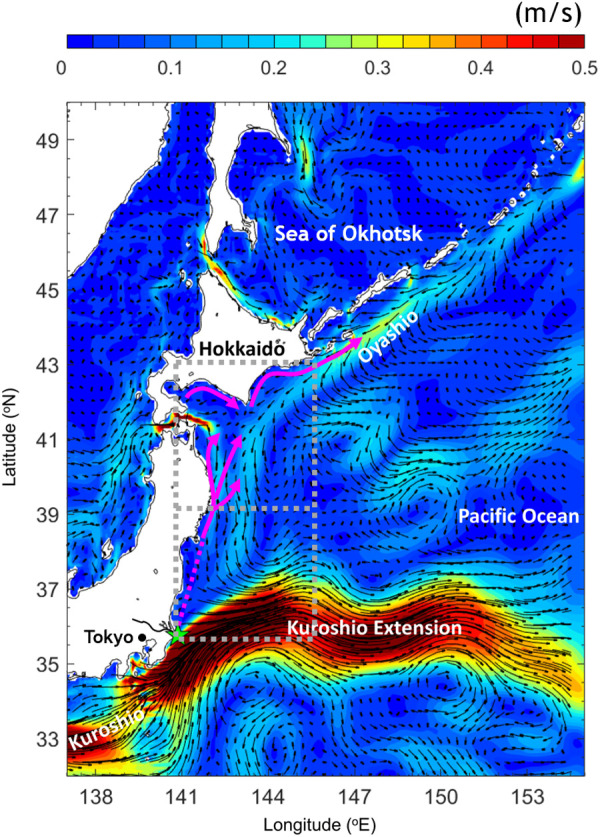
Map of the study area and mean ocean currents over the tracked period (mid-February–mid-July 2001 to 2020 at a depth of 5 m. Possible migration routes of chum salmon (*Oncorhynchus keta*) from the Tone River are plotted in magenta, where the solid line is taken from [20] and the dashed line is projected. The color shading represents the current speed (m/s), and the current vectors are superimposed. The green star shows the location of the Tone River mouth where virtual chum salmon fry were released. The black curve next to the river mouth shows the major Tone River water line. The gray-dashed boxes show the defined (both boxes) and early (southern box) migration areas. *This figure was created using MATLAB R2021a (*http://www.mathworks.com/*).*

Chum salmon are semelparous fish that spawn in rivers during fall and winter, and migrate to the sea the following spring [7]. After descending natal rivers in Japan, chum salmon fry move northward in the ocean to appear in large numbers along the coast of Hokkaido from spring to early summer. They then enter the Sea of Okhotsk, where they spend their first summer and fall (Fig 2) [12]. While salmon populations are also declining in the rivers of Hokkaido, the rate of decline has not been as severe as that observed in the Tone River. This discrepancy suggests that the decline may be attributed to the northward migration process from the Tone River to the Hokkaido coast in spring and early summer. If early mortality is a key factor, mortality rates may have increased significantly in recent years during the northward migration of chum salmon fry from the Tone River toward the Hokkaido coast.

In this study, we examined the factors that prevent chum salmon fry from migrating northward from the southern limit of their distribution. We hypothesized that there are three potential factors: ocean currents, water temperature, and zooplankton. Chum salmon fry have limited swimming ability, and previous studies have identified ocean currents as a factor that influences salmon migration [13,14]. The ocean currents off the Pacific coast of northern Japan are particularly complex (Fig 2). The Kuroshio, which is characterized by warm temperature and low nutrient content, flows northeastward along the Pacific coast of southern Japan and turns eastward to form the Kuroshio Extension [15]. In contrast, the Oyashio originates in the subpolar Pacific and carries cold water southward along the eastern coast of Hokkaido. The confluence of these currents off the Pacific coast of northeastern Japan creates a complex hydrodynamic system involving water from the Kuroshio and Kuroshio Extension transported by eddies and the southward-flowing Oyashio [16]. The southward-flowing Oyashio can hinder the northward migration of chum salmon fry toward Hokkaido, and the mesoscale eddies in this region may trap chum salmon fry and potentially affect their dispersal and survival [17]. Notably, the mouth of the Tone River is located at the edge of the Kuroshio/Kuroshio Extension, which may prevent chum salmon fry from migrating northward and instead carry them far to the east. In recent decades, the Kuroshio/Kuroshio Extension and Oyashio had been observed to shift northward [18,19]. These shifts in ocean currents caused by climate change can raise difficulties in the northward migration of salmon fry.

The mortality rates of chum salmon fry may also have been increased by high water temperatures. The distribution of chum salmon fry during early marine life is influenced by the water temperature, which affects their growth rate and feeding efficiency [21]. Laboratory experiments and field surveys have shown that the optimal water temperature for chum salmon is 8°C–13°C [21–23] while they can tolerate 5°C–24°C [24]. The decline of the chum salmon population in Japan may have been influenced by warming water temperature [25], which have been shown to have a great impact on marine ecosystems [26]. Marine heatwaves have occurred along the Oyashio region since 2010 [27], and the increased water temperatures may harm chum salmon fry during their migration [25].

Finally, zooplankton are the main food source of chum salmon fry, whose growth rate is affected by food availability. A high abundance of zooplankton supports the growth of chum salmon fry during their early marine life [28]. Changes in ocean currents and water temperature can alter zooplankton abundance and composition. In recent years, the northward-flowing warm Kuroshio has been strengthening while the southward-flowing cold Oyashio has been weakening [18]. The Oyashio is richer in nutrients and thus increases food availability compared with the nutrient-poor Kuroshio [29–31]. Declines in zooplankton abundance associated with changes in ocean currents may limit food availability for chum salmon fry and thus increase mortality rates due to starvation and starvation-related causes.

In this study, we investigated the factors behind the recent decline of the chum salmon population in the Tone River by examining the effects of the three above factors. A 20-year (2001–2020) simulation of the dispersal of virtual salmon fry released at the mouth of the Tone River was conducted by using a particle-tracking model that incorporated transport by ocean currents and active swimming behavior. We used the results of this numerical simulation along with reanalysis data on water temperature and zooplankton biomass to identify the key factors contributing to the recent population decline of chum salmon at the southern limit of their distribution.

## Materials and methods

### Chum salmon data

The number of chum salmon migrating to the Tone River is monitored at three fish ladders located at a weir (Tone Oozeki; 36.19° N, 139.47° E) approximately 150 km upstream from the river mouth (Fig 1). Since 1983, counts have been conducted from October to December, and the data have been made publicly available by the Japan Water Agency (https://www.water.go.jp/kanto/tone/water/fish-data/) [32]. Although fishermen have conducted hatchery stocking programs in tributaries downstream of this weir where they join the Tone River, chum salmon home toward natal rivers at the tributary level [33,34], so information from other tributaries was not considered.

Chum salmon spawning in rivers occurs during fall and winter. Salmon fry migrate to the sea the following spring, and adults return to their natal rivers 3–5 years later during fall [7]. Surveys conducted in 2003, 2004, 2017, and 2018 identified 4-year-old fish as the predominant age class among chum salmon captured at Tone Oozeki, irrespective of the survey year [35]. In this study, the population growth rate of chum salmon that enter the ocean in year *t* (PGR*t*) was calculated under the assumption that one generation comprises 4 years because 4-year-old fish are the dominant age group of this population:


$${\text{PGR}}_{t}=\text{ln }\left(N_{t+\text{3}}\right)-\text{ln }\left(N_{t-\text{1}}\right)$$ (1)

where *N*_*t*_ is the number of adult chum salmon counted in year *t*. PGR*t* represents the survival rate of fry that spawned in year *t* − 1, descended to the sea in the following spring of year *t*, and returned as adults in year *t* + 3 and thus approximates the intrinsic rate of increase in population per generation.

### Ocean reanalysis data

The Japan Coastal Ocean Predictability Experiment 2 (JCOPE2M) is a data-assimilative ocean circulation model that was developed by the Japan Agency for Marine-Earth Science and Technology and provided the three-dimensional hydrological fields including ocean currents and water temperatures for particle-tracking experiments and quantifying environmental conditions in both this study and previous studies [19,36–39]. The model domain of JCOPE2M encompasses the western North Pacific (10.5°–62° N, 108°–180° E) with a horizontal resolution of 1/12° (8–9 km) and 46 vertical layers. The external forcings that drive JCOPE2M include wind stresses and net heat/freshwater fluxes at the sea surface, which are converted from the six-hourly atmospheric reanalysis products of the National Centers for Environmental Prediction/National Center for Atmospheric Research. JCOPE2M includes 108 major rivers in the western Pacific are included. Satellite data on the sea surface temperature, sea surface height, in situ temperature, and salinity are assimilated into JCOPE2M by a multiscale three-dimensional variational method [40]. A previous study [36] showed that JCOPE2M performed adequately in simulating the observed three-dimensional circulation of the western North Pacific. Data from JCOPE2M are available from 1993 to the present.

The zooplankton biomass data were taken from the Low and Mid-Trophic Levels reanalysis product for the global ocean [41], which provides two-dimensional fields of the biomass content in the epipelagic and upper and lower mesopelagic layers. The mass content of zooplankton expressed as carbon in seawater (g/m^2^) was used to represent the zooplankton biomass. The model has a horizontal resolution of 0.083°, and data are available from January 1998 to December 2023.

To serve as explanatory variables for the population growth rate of chum salmon, the mean zooplankton biomass and temperature at a depth of 5 m were calculated from mid-February to mid-July for each year across the potential migration area after leaving estuaries, which was defined as between the mouth of the Tone River and southern Hokkaido (35.79°N–43° N, 140.8°E–146° E). Notably, the actual migration area is unknown. The potential migration area defined herein referred to the longitude and attitude between the mouth of the Tone River and the eastern edge of Hokkaido (Fig 2). Because early starvation and nutritional deficiency are potential causes of mortality in marine fish species [42], the initial zooplankton availability during the early migration period (mid-February–end of April) in the early migration area (35.79°–39.4° N, 140.8°–146° E, Fig 2) was also calculated for each year. Similarly, the initial water temperature during the early migration period and across the early migration period area defined above was calculated on an annual basis to compare it with the zooplankton data.

### Particle-tracking simulation

A particle-tracking simulation was conducted to evaluate the effects of ocean currents and swimming behavior on the migration of chum salmon fry from the Tone River to Hokkaido. The particle-tracking scheme developed by Ohashi and Sheng [43] based on the fourth-order Runge–Kutta method [44] was used. The tracking and output time step was set to 3 h. Random walk displacement was included to represent the unresolved sub-grid turbulent flow [43]. The estimated maximum horizontal and vertical displacements due to random walk were 600 and 20 m, respectively.

Numerical simulations were conducted to investigate the variation in chum salmon fry migration over the 20-year period of 2001–2020. Virtual salmon fry (v-salmon fry) were released at the mouth of the Tone River (35.76°–35.96° N, 140.86°–141.06° E) at different locations separated by 3 km in the zonal and meridional directions. The release depth was set at 5 m based on a previous study that observed chum salmon fry near Hokkaido at a water depth of 0–6 m [23]. Chum salmon fry have been observed to descend the Tone River and a nearby river from mid-February to mid-April [45,46], so the release times were set to this period each year at intervals of 10 days. Chum salmon fry have been observed to arrive near Hokkaido between late May and mid-July [20,47], so the tracking period was set to 100 days and ended on July 15.

In this study, the horizontal swimming behavior of chum salmon fry was considered. Because the actual swimming behavior is unclear, several swimming strategies were designed based on the known information from previous studies. The initial body length was set at 5 cm based on the observed body length of chum salmon fry in this region [45,46]. The growth rate was set to 0.65 mm/day based on otolith analysis of chum salmon collected in northeastern Japan [47]. The default swimming speed was set to 2 body length per second (BL/s), which appears to be a possible cruising speed for chum salmon fry [12,48]. The swimming direction was determined by the relative direction between the starting point and destination, where 0° was defined as true north and the angle increased clockwise. Positive and negative angles with absolute value <90° were northeastward and northwestward, respectively. In the numerical simulations, six different scenarios were considered with most variables kept the same except for the swimming strategy (Table 1): In Scenario 1, the swimming direction was randomly set between − 5° (northwest) and 25° (northeast). The swimming direction was fixed at 20° (northeast) in Scenario 2. In Scenario 3, the v-salmon fry searched for colder water, so the swimming direction could vary at each time step. We also considered that chum salmon fry may not start swimming immediately after leaving the river. Nagata [22] previously showed that the offshore movement of chum salmon fry began when their body length reached about 7 cm, which was about 30 days after departure in our simulation. To account for this factor, Scenario 4 set the v-salmon fry to swim passively for the first 30 days and then the swimming direction was fixed to − 20° (northwest). In Scenario 5, the v-salmon fry tried to stay near the starting point for the first 30 days even if they were swept away by the ocean currents; then, the swimming direction was fixed at − 15° (northwest). Finally, Scenario 6 examined the case of slower swimming at a speed of 1 BL/s while the swimming direction was fixed at 20° (northeast).

**Table 1:** Parameters used in the numerical simulations of virtual salmon fry tracking and the estimated migration durations to southern Hokkaido under the six scenarios. Positive and negative angles used in the swimming direction denote northeastward and northwestward, respectively.

Starting point	Tone River mouth (35.86°N ± 0.1°N, 140.96°E ± 0.1°E)			
Destination	Southern Hokkaido (41°N–43°N, 141°E–146°E)			
Period	2001–2020			
Release dates	21 Feb, 1 Mar, 11 Mar, 21 Mar, 1 Apr, 11 Apr			
Tracking period	100 days			
Tracking depth	5 m			
Swimming strategies	Scenario	Swimming direction	Swimming speed	Duration (days)
	1	Random between −5°and 25°	2 BL/s	69.0 ± 7.6
	2	20°	2 BL/s	69.3 ± 7.1
	3	Swim toward colder water	2 BL/s	80.3 ± 6.9
	4	Passive for first 30 days then −20°	2 BL/s	74.9 ± 6.6
	5	Try to stay near the starting point for first 30 days then −15°	2 BL/s	76.3 ± 6.6
	6	20°	1 BL/s	79.0 ± 6.8

To quantify the degree of successful northward movement, the arrival rate in southern Hokkaido was calculated as the number of v-salmon fry that reached their destination (41°N–43° N, 141°E–146° E) divided by the total number of v-salmon fry released in each scenario. Additional conditions were incorporated to analyze the simulation results when v-salmon fry encountered water masses exceeding specific temperature thresholds during their migration. Previous field studies have shown that chum salmon fry were not found in waters warmer than 13°C [22]. Recent experimental studies have indicated that the optimal water temperature for chum salmon in terms of growth efficiency and metabolic cost of swimming is up to 13°C [21,49]. Accordingly, the lethal temperature threshold was set to 13°C. However, freshwater experiments conducted under controlled conditions have demonstrated that chum salmon fry can survive at much higher temperatures of up to 24°C [24]. In saltwater rearing experiments, the preferred temperature range has been reported to be 13°C–18°C [50] with the growth rate maximized at 16°C [21]. Therefore, 16°C was used as an alternative threshold. Thus, additional arrival rates were calculated under the assumption that mortality occurred when the temperature exceeded either 13°C or 16°C.

The visitation frequency, describing the distribution of v-salmon fry, was defined as the number of v-salmon fry reaching an individual grid. For each v-salmon fry, all grids it passed through were counted once, regardless of the duration within the grid.

### Statistical analysis

In this study, the population growth rate (PGR_t_) of chum salmon in the Tone River was the variable of interest and was analyzed in relation to several quantitative explanatory variables. Six scenarios of arrival rates derived from particle-tracking simulations were considered that represented the probability that a salmon fry descending from the Tone River in spring would reach Hokkaido through complex ocean currents. Two lethal temperature thresholds were applied as supplementary conditions to the arrival rates. Four environmental variables were extracted from the oceanographic reanalysis data: the mean water temperature across the potential migration area, zooplankton biomass over the same area, the initial zooplankton availability and initial temperature in the early migration area. Pearson’s correlation coefficients were used to examine the relationships among variables. Because the water temperature and zooplankton availability are interrelated, a partial correlation analysis was conducted to disentangle their individual effects.

## Results

**Fig 3:**
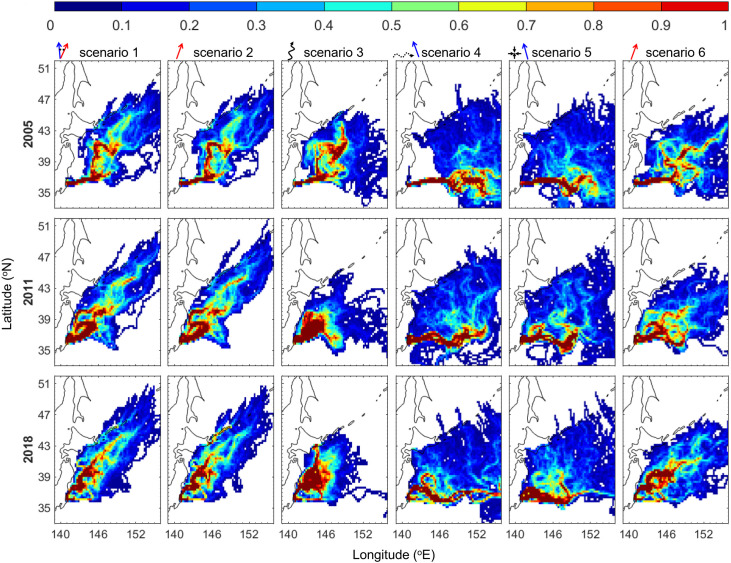
Normalized visitation frequency of v-salmon fry from the Tone River in different simulation scenarios: 2005 (top), 2011 (middle), and 2018 (bottom). Trajectories and arrows on the top of each panel illustrate the v-salmon fry swimming directions used in each scenario. *This figure was created using MATLAB R2021a (*http://www.mathworks.com/*).*

The population growth rate of chum salmon in the Tone River was generally positive before 2010 (Fig 1b). The population growth rate became negative in 2011 and remained below the minimum value before 2010 since 2013. The visitation frequency was calculated to determine the v-salmon fry migration distribution for all scenarios (Fig 3). The visitation frequency was widely distributed off the northeast coast of Japan in all scenarios with different swimming strategies. In all scenarios, the v-salmon fry arrived in southern Hokkaido, although their distributions differed. In Scenarios 1 and 2, v-salmon fry with directional swimming exhibited less eastward dispersion compared with those in the other scenarios. V-salmon fry without directional swimming searching for cold water (Scenario 3) exhibited the least northward distribution compared with those of the same swimming speed in the other scenarios. In Scenarios 4 and 5, wherein directional swimming was not assumed during the early migration period, v-salmon fry were transported eastward by the Kuroshio Extension before turning northward with directional swimming. The slower swimming speed assumed in Scenario 6 resulted in a less northward and more eastward distribution compared with that in Scenario 2, where the same swimming strategy and a higher swimming speed were assumed. The years 2005, 2011, and 2018 were selected because they correspond to the years when the population growth rate was highest, first became negative, and was lowest during the study period, respectively. Based on the population growth rate (Fig 1b), more v-salmon fry were expected to arrive in southern Hokkaido in 2005 and 2011 than in 2018, but the simulation results did not show a clear difference between these three years regardless of the scenario (Fig 3).

**Fig 4:**
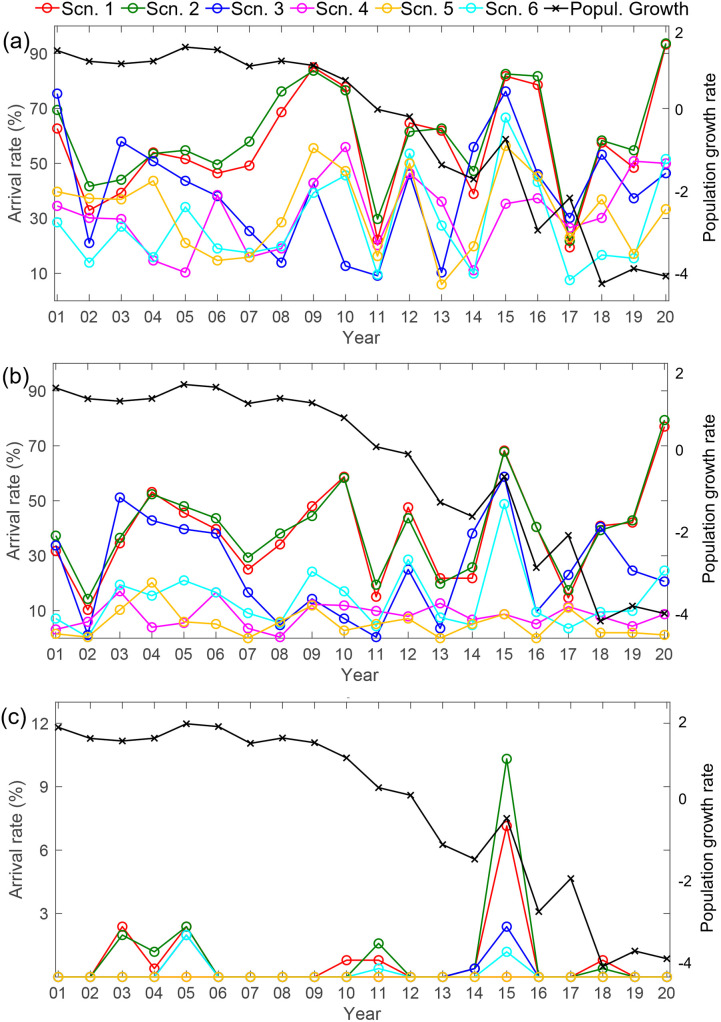
Arrival rates (%) of v-salmon fry from the Tone River mouth at southern Hokkaido (41°N–43°N, 141°E–146°E) from 2001 to 2020 with different lethal temperature thresholds: (a) no threshold, (b) 16°C, and (c) 13°C. The red, green, blue, magenta, gold, and cyan lines represent Scenarios 1–6, respectively. The black line shows the population growth rate of chum salmon. *This figure was created using MATLAB R2021a (*http://www.mathworks.com/*).*

The arrival rates of v-salmon fry to southern Hokkaido were positively correlated among the six scenarios (mean pairwise correlation, *r* = 0.50, S1 Table) and show no sign of a decreasing trend over the 20-year study period (Fig 4a). They were not correlated with the population growth rate of chum salmon regardless of the scenario (*r* = −0.32 to 0.10, all *p* > 0.05, S2 Table). This indicates that the hypothesis that the decline of chum salmon in the Tone River attributed to changes in ocean currents is not supported. Even with the lethal temperature thresholds, the arrival rates of v-salmon fry were uncorrelated with the population growth rate of chum salmon (Fig 4b and 4c; *r* = −0.17 to 0.33, all *p* > 0.05, S2 Table), with the highest positive correlation observed in Scenario 5 under the 16°C threshold. This indicates that the hypothesis that the decline of southern limit salmon populations caused by chum salmon fry encountering high water temperatures above the lethal temperature threshold is not supported.

**Fig 5:**
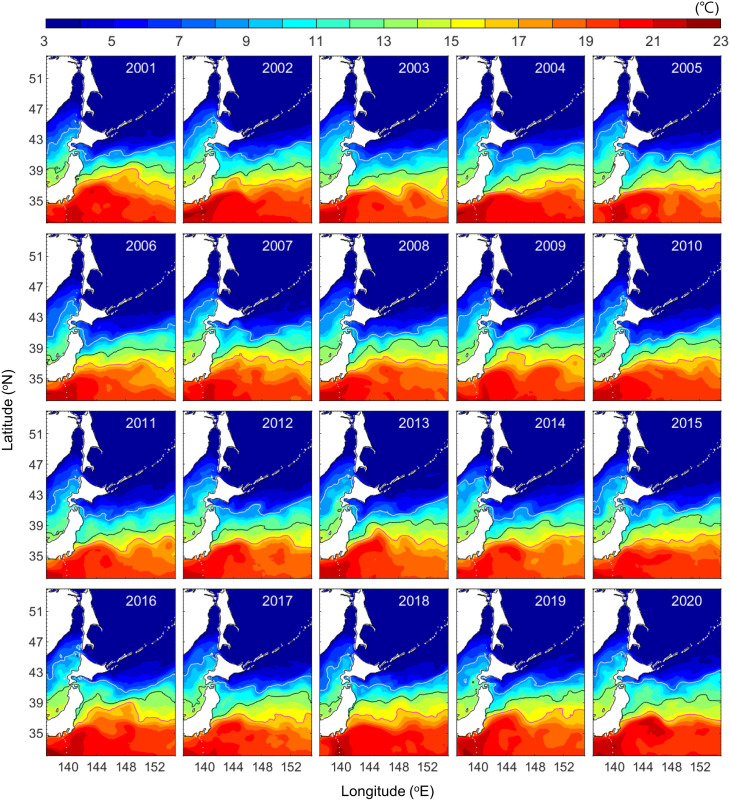
Mean water temperature from mid-February to mid-July at a depth of 5 m for the years of 2001–2020. The magenta, black, and white contours represent the 16°C, 13°C, and 8°C isotherms, respectively. *This figure was created using MATLAB R2021a (*http://www.mathworks.com/*).*

**Fig 6:**
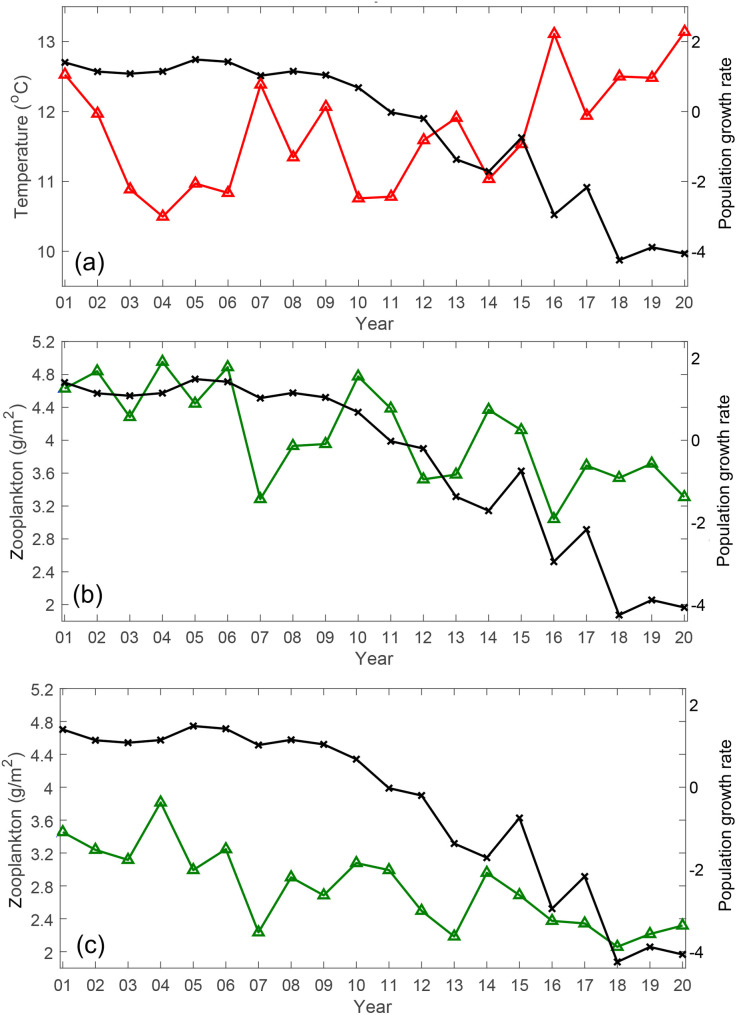
Time series of potential factors affecting chum salmon fry migration. Mean (a) water temperature (°C) and (b) zooplankton biomass (g/m^2^) from mid-February to mid-July over the migration area (35.8°N–43° N, 140.8°E–146°E) for the years 2001–2020; (c) initial zooplankton availability (g/m^2^) for the early migration period (mid- February to end of April) and initial migration area (35.8°N–39.4°N, 140.8°E–146°E) for the years 2001–2020. The black lines indicate the population growth rate of chum salmon. *This figure was created using MATLAB R2021a (*http://www.mathworks.com/*).*

Given the decline in population growth, there must be a cause, even if it is not ocean currents or lethal temperatures. The water temperature not only affects mortality but also the metabolic rate and food availability. The average water temperature from mid-February to mid-July in the migration area has changed over the last 20 years (Fig 5). The 16°C isotherm is near the mouth of the Tone River while the 8°C isotherm is north of southern Hokkaido. In 2010, the 8°C isotherm extended southward to about 39°N while the 16°C isotherm was at about 36° N. In 2020, the 8°C isotherm retreated northward to about 42°N while the 16°C isotherm shifted north to about 37° N. The time series of the mean temperature during migration (mid-February to mid-July) in the ocean region extending from the mouth of the Tone River to southern Hokkaido was negatively correlated with the population growth rate of chum salmon (Fig 6a; *r* = −0.61, *p* = 0.005).

**Fig 7:**
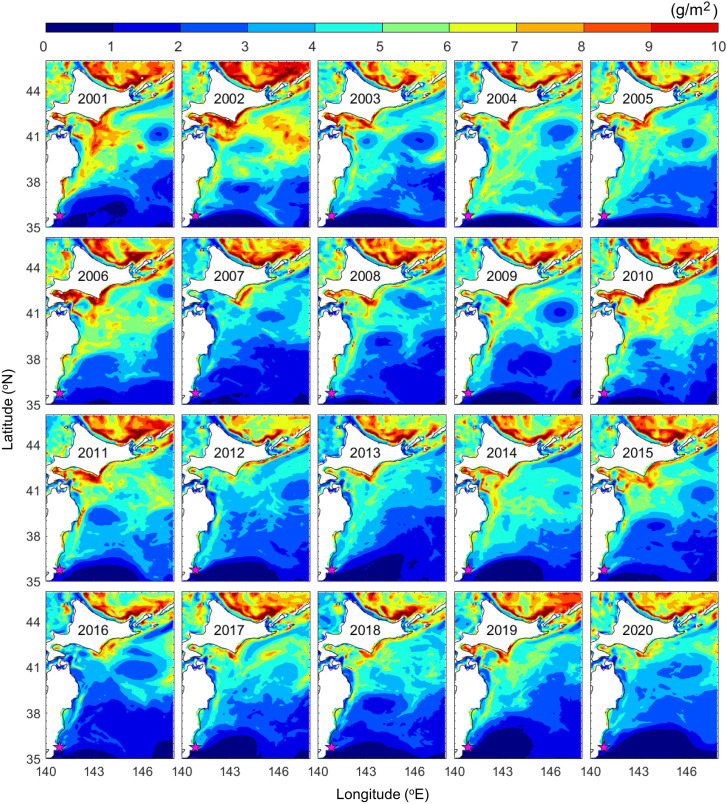
Mean zooplankton biomass (g/m^2^) in the migration area from mid-February to mid-July for the years 2001–2020. *This figure was created using MATLAB R2021a (* http://www.mathworks.com/ *).*

The zooplankton distribution during the study period showed a lower zooplankton biomass in the region of the Kuroshio and Kuroshio Extension than off southern Hokkaido (Fig 7). The low-zooplankton region (<1 g/m^2^) reached its southernmost location (~35°N) in 2006 and then gradually moved northward above 37° N after 2019. The time series of the mean zooplankton biomass during migration (mid-February to mid-July) in the area between the mouth of the Tone River and southern Hokkaido showed a decreasing trend over the last 20 years (Fig 6b) and had a significant correlation with the population growth rate of chum salmon (*r* = 0.65, *p* < 0.01).

**Fig 8:**
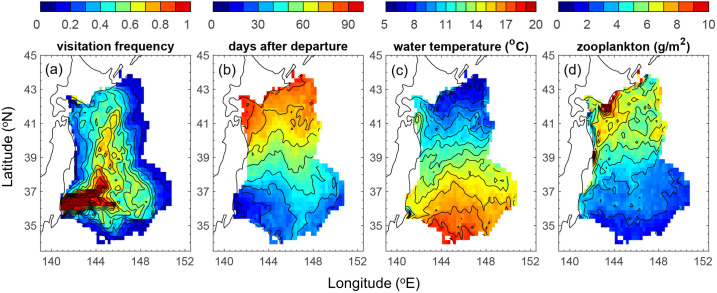
20-year composite trajectories of the v-salmon fry that reached Hokkaido based on Scenario 5 (a) visitation frequency, (b) days after departure, (c) temperature along migration route, (d) zooplankton abundance along migration route. This figure was created using MATLAB R2021a (http://www.mathworks.com/).

Additionally, we examined the time-varying migration process. Using Scenario 5, which exhibited the highest correlation with the population growth rate as an example, we composite the visitation frequency, duration, along-track temperature and zooplankton abundance for the v-salmon fry that reached southern Hokkaido (Fig 8). During the first 30 days, most v-salmon fry were carried eastward by the Kuroshio Extension before turning north toward Hokkaido (see Fig 8a and 8b). The temperature experienced along the migration route in the Kuroshio Extension was 15°C –16°C and gradually decreased to <10°C upon reaching Hokkaido (Fig 8c). The zooplankton abundance along the route was generally low (<3 g/m^2^) in the Kuroshio Extension during the first half of the journey (Fig 8d). It then increased northward, reaching a local maximum of >10 g/m^2^ near Hokkaido. Reportedly, v-salmon fry remained in the Kuroshio Extension during the first half of the simulation, wherein the water was relatively warm and zooplankton abundance was generally low, indicating that it could be critical for early salmon fry migration. Therefore, we repeated the correlation analysis of temperature and zooplankton against the salmon population growth rate by focusing on the Kuroshio Extension and considering only the first half of the migration period. The initial zooplankton availability showed a higher correlation with population growth rate (Fig 6c; *r* = 0.73, *p* < 0.001) than that of the entire journey. However, the correlation between the initial temperature and population growth rate was decreased (*r *= −0.50, *p* = 0.026).

**Fig 9:**
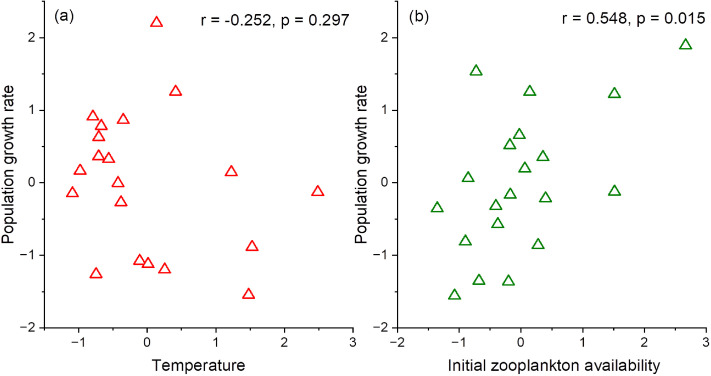
Partial correlation analysis on the effects of the (a) water temperature and (b) initial zooplankton availability on the population growth rate of chum salmon. Each panel shows the relationship between standardized residuals from linear regressions in which the influence of the other variable was controlled. For the water temperature effect, the residuals were obtained by regressing the temperature and population growth rate separately on zooplankton (control variable). For zooplankton effect, residuals were obtained by regressing the zooplankton availability and population growth rate separately on the water temperature (control variable). This figure was created using OriginPro2025 (https://www.originlab.com/).

The above results indicate that the water temperature and zooplankton availability (i.e., food source) influence the population growth rate of chum salmon. However, the water temperature and the zooplankton availability are interrelated. Partial correlation analysis revealed that the water temperature did not have a significant correlation with the population growth rate when the effect of initial zooplankton availability was controlled for (Fig 9a; *r* = −0.25, *p* = 0.297). In contrast, the initial zooplankton availability retained a significant correlation with the population growth rate even after the effect of the water temperature was controlled for (Fig 9b; *r* = 0.55, *p* = 0.015).

## Discussion

In this study, we investigated the causes of the recent decline in chum salmon population of the Tone River using ocean reanalysis data and particle-tracking simulations from 2001 to 2020. The simulation released v-salmon fry at the mouth of the Tone River during each year with six different combinations of swimming strategies to attempt to evaluate the ocean circulation that might influence the population growth rate of chum salmon. The simulation results could not reproduce the observed decline in population regardless of the selected swimming strategy and addition of lethal temperature thresholds, which indicated that ocean currents and lethal water temperatures are not significant factors. Furthermore, we examined additional lethal temperatures of 20°C (results not shown). However, the water temperatures in the migration region rarely exceeded 20°C, and the arrival rates were similar to those when no lethal temperature threshold was applied. Consequently, the conclusions remained unchanged regardless of the lethal temperature value used. In recent decades, the Kuroshio and Kuroshio Extension currents have shifted northward which would weaken the southward current in the migration area [18,19] and dynamically help transport v-salmon fry northward, which would explain the poor correlation of the simulation results with the salmon population decline.

The northward shifts of the Kuroshio/Kuroshio Extension and Oyashio were accompanied by an increase in the average water temperature in northeastern Japan. The northward shift of the nutrient-poor Kuroshio/Kuroshio Extension also reduced the zooplankton biomass in this area [29]. Zooplankton from the Oyashio is an important food source for chum salmon fry, and its abundance affects their growth and survival [28,30,31]. The decline in the salmon population growth rate was correlated with increase in water temperature and the reduced zooplankton availability. However, when partial correlation analysis was applied, the water temperature was no longer important; only the zooplankton remained significant. The results indicated that the initial zooplankton availability is the only factor with a significant correlation to the population growth rate of chum salmon. Because early starvation and nutritional deficiency have been suggested as major causes of mortality for marine fish species [42], the decline of chum salmon at the southern limit of their range is more likely to be directly related to a lack of food rather than an increase in water temperature. Changes in food availability and their impact on salmon fry have been observed elsewhere [28,30]. In western Alaska, climate change has increased the proportion of low-quality prey with less energy density for chum salmon fry [51].

Different swimming strategies resulted in different migration distributions in the simulations. The actual swimming strategy of chum salmon fry is unclear, and it is also unknown which simulated migration distribution is closest to reality. However, scenarios 4 and 5 more closely captured the observed arrival of chum salmon fry in southwestern Hokkaido (west of 143°E) [20] while the other scenarios resulted in chum salmon fry only arriving in eastern Hokkaido (east of 143°E). With the default swimming speed of 2 BL/s (scenarios 1–5), the migration from the mouth of the Tone River to southern Hokkaido took approximately 69–80 days, which matches observations of salmon fry arriving along the Hokkaido coast in June [20,47]. Scenario 6 corresponded to scenario 2 except it used a slower swimming speed of 1 BL/s, which increased the duration of the migration by ~10 days. Although the slow 1 BL/s swimming speed resulted in a later arrival to Hokkaido compared with the high 2 BL/s speed, the v-salmon fry that departed from the Tone River during mid-March reached Hokkaido in June, indicating that both speeds are reasonable for simulation. Additionally, the two scenarios with different swimming speeds resulted in similar variations in the arrival rates; however, the actual rates considerably differ between the two scenarios. The 1BL/s swimming speed was affected more by the ocean currents than the 2 BL/s speed, resulting in a less effective northward migration than the 2 BL/s speed and thus, in a relatively low arrival rate. Although the number of v-salmon fry released each time was fixed, the actual migration amount could vary with time. Different release settings may affect the arrival rates and distributions. The uncertainty over the swimming strategy and migration distribution did not affect the explanation of salmon population decline because the arrival rates were positively correlated among the scenarios; additionally, the ocean currents did not play a crucial role.

If the reduced zooplankton availability impacts the chum salmon population, the northward shift of the Kuroshio/Kuroshio Extension should affect not only the chum salmon population at Tone River but also at rivers between Tone River and southern Hokkaido. In fact, chum salmon populations in the northeastern region of Japan’s main island (Honshu) have been in a severe decline since 2010 [52]. Satellite observations indicate that the Kuroshio/Kuroshio Extension have shifted northward since 2007 [18], but they also exhibit a decadal variability on a time scale of ~10 years [53]. The northward shift of the Kuroshio/Kuroshio Extension may reach an inflection point in the coming few years that will hopefully mitigate the decline of the chum salmon population, but this prospect remains uncertain because the Kuroshio has been projected to move further northward until the end of the century [54].

In this study, we focused on the early mortality of chum salmon fry in the first few months after they entered the ocean. Under climate change, the northward shifts of the Kuroshio/Kuroshio Extension and Oyashio appear to be causing a critical food shortage for chum salmon fry migrating northward from the Tone River. However, climate change is also likely to impact chum salmon adults by reducing growth and maturity rates [55,56] and increasing their exposure to high water temperature when they return to their natal rivers, which may prevent them from reaching their spawning sites [10,57]. In addition, the in-river spawning and survival of newborn fry may be particularly sensitive to warming water temperature at the southern limit of their distribution [58]. A comprehensive understanding of these impacts remains an important challenge for future research.

## Supporting information

S1 Table Pairwise correlation coefficients of the arrival rates of v-salmon fry to southern Hokkaido across six scenarios. (PDF)

S2 Table Correlation coefficients between the arrival rates of v-salmon fry and the population growth rate (PGR) of chum salmon under different lethal temperature thresholds. (PDF)
