# Supplementary material for: Beyond lethal temperatures: Factors behind the disappearance of chum salmon from their southern margins under climate change
Source: PLoS One. 2025 Sep 10;20(9):e0330957. doi: 10.1371/journal.pone.0330957 (PMC12422446; doi:10.1371/journal.pone.0330957)
Supplement: S1 Table — (PDF) [file pone.0330957.s001.pdf]

S1 Table Pairwise correlation coefficients of the arrival rates of v-salmon fry to southern Hokkaido across six scenarios

Pearson's *r*	Scn. 1	Scn. 2	Scn. 3	Scn. 4	Scn. 5	Scn. 6
Scn. 2	0.986**					
Scn. 3	0.255	0.246				
Scn. 4	0.527*	0.476*	-0.001			
Scn. 5	0.565**	0.516*	0.479*	0.358		
Scn. 6	0.808**	0.761**	0.359	0.544*	0.646**	

* $P < 0.05$, ** $P < 0.01$
