# Supplementary material for: Beyond lethal temperatures: Factors behind the disappearance of chum salmon from their southern margins under climate change
Source: PLoS One. 2025 Sep 10;20(9):e0330957. doi: 10.1371/journal.pone.0330957 (PMC12422446; doi:10.1371/journal.pone.0330957)
Supplement: S2 Table — (PDF) [file pone.0330957.s002.pdf]

S2 Table Correlation coefficients between the arrival rates of v-salmon fry and the population growth rate (PGR) of chum salmon under different lethal temperature thresholds

No thresho	Scn. 1	Scn. 2	Scn. 3	Scn. 4	Scn. 5	Scn. 6
PGR	-0.143	-0.126	-0.11	-0.321	0.102	-0.048
16°C	Scn. 1	Scn. 2	Scn. 3	Scn. 4	Scn. 5	Scn. 6
PGR	-0.174	-0.144	0.003	0.047	**0.325**	0.050
13°C	Scn. 1	Scn. 2	Scn. 3	Scn. 4	Scn. 5	Scn. 6
PGR	0.079	0.065	0.109	na	na	0.195

na = scenario excluded due to all arrival rates being zero.
